# Needle-in-needle micropuncture technique for epicardial access during ventricular tachycardia ablation

**DOI:** 10.1007/s10840-026-02400-z

**Published:** 2026-07-21

**Authors:** Roland Richard Tilz, Roman Mamaev, Sascha Hatahet, Tuğba Aktemur Özalp, Sorin Stefan Popescu, Jan-Per Wenzel, Mirco Küchler, Charlotte Eitel, Karl-Heinz Kuck, Kohei Ukita

**Affiliations:** 1 Department of Rhythmology, University Heart Center Lübeck, University Hospital Schleswig-Holstein, Lübeck, Germany; 2 German Center for Cardiovascular Research (DZHK), Partner Site Lübeck, Germany; 3 Department of Rhythmology, University Heart Center, University Hospital Schleswig-Holstein, Ratzeburger Allee 160, Lübeck, 23562 Germany

**Keywords:** Catheter ablation, Complete atrioventricular block, Epicardial approach, Micropuncture technique, Ventricular tachycardia

## Abstract

**Background:**

Epicardial access is often required for catheter ablation of ventricular tachycardia (VT) when arrhythmogenic substrates are located on the epicardial surface, but it is associated with potentially serious complications, including cardiac tamponade and coronary artery injury. The use of a needle-in-needle (NiN) micropuncture technique for percutaneous epicardial access may reduce myocardial trauma and improve procedural safety. We aimed to describe a standardized workflow and assess the feasibility and acute safety of epicardial access for VT ablation using a NiN micropuncture technique.

**Methods:**

This retrospective, single-center observational study without a comparator group included 40 consecutive patients who underwent epicardial access for VT ablation using the NiN micropuncture technique between January 2020 and July 2025. Baseline clinical characteristics, procedural details, success of epicardial access, and acute access-related complications were evaluated.

**Results:**

Nurse-administered propofol sedation was used in 37 patients (93%). The median total procedure time was 141 min. Epicardial access was successfully achieved in all patients (100%). No major access-related complications occurred, including cardiac tamponade, significant bleeding, or coronary artery injury. One patient developed complete atrioventricular block during epicardial ablation, which was unrelated to the access technique. All patients were discharged without procedure-related disability or death.

**Conclusion:**

Epicardial access using a NiN micropuncture technique was feasible and acutely safe for VT ablation, achieving a 100% access success rate with no major puncture-related complications.

## Introduction

Catheter ablation of ventricular tachycardia (VT) often requires percutaneous epicardial access when arrhythmogenic substrates are located on the epicardial surface or within the myocardium [1]. Despite its widespread adoption, gaining access to the pericardial space remains technically demanding and carries a risk of serious complications. Even in experienced centers, major complications related to epicardial VT ablation procedures have been reported in approximately 5–10% of cases [2–4]. Importantly, complications reported during epicardial VT ablation include both access-related events (such as bleeding or organ injury) and complications related to epicardial mapping or ablation itself.

One promising refinement is the use of a small-gauge (21-gauge [21 G]) micropuncture needle instead of the conventional large-bore (17–18 G) epidural needle. Reduced insertion force may confer greater tactile feedback and control, decreasing the risk of deep myocardial penetration once the pericardial space is entered. However, standard micropuncture approaches may still be limited by insufficient needle support, difficulty in traversing fibrotic pericardium, and challenges in maintaining precise needle control.

To address these limitations, a needle-in-needle (NiN) micropuncture technique has been developed, combining the stability of an outer guiding needle with the atraumatic profile of an inner fine-gauge needle [5, 6]. This coaxial approach allows controlled advancement toward the pericardial space while minimizing the risk of excessive myocardial penetration. By providing enhanced directional control and stepwise access, the NiN technique may facilitate safer epicardial puncture even in anatomically challenging cases.

In this study, we describe the standardized workflow of the NiN micropuncture technique for epicardial access during VT ablation and evaluate its feasibility and safety in a contemporary cohort.

## Methods

### Study design and population

This retrospective, single-center observational study included patients who underwent epicardial approach during catheter ablation for drug-refractory VT using a NiN micropuncture technique between January 2020 and July 2025 in our institution. All epicardial accesses performed for VT ablation during this study period were performed using the NiN micropuncture technique by a single experienced operator. No conventional large-bore needle epicardial access cases were performed during the same study period. The endpoint of this study was feasibility of epicardial access and occurrence of major access-related complications defined as cardiac tamponade requiring intervention, coronary artery injury, emergency cardiac surgery, abdominal organ injury, phrenic nerve injury, pneumothorax, need for surgical repair, blood transfusion, or procedure-related hemodynamic compromise.

The study protocol was approved by the local institutional review board, and all patients provided informed consent for the procedure in accordance with standard practice.

### Ablation procedure and epicardial access

The ablation procedure was carried out under nurse-administered propofol sedation or general anesthesia per institutional protocol. Patients underwent nurse-administered propofol sedation when adequate procedural tolerance was anticipated. General anesthesia was selected when airway control was considered necessary or according to procedural complexity. An anesthesia team was available for support.

Diagnostic catheters were placed in the coronary sinus and right ventricle. In patients who underwent an endocardial approach, a mapping catheter was advanced into the right ventricle (transvenous approach), left ventricle (transseptal approach or retrograde access), or both, depending on the presumed ablation target.

**Fig. 1:**
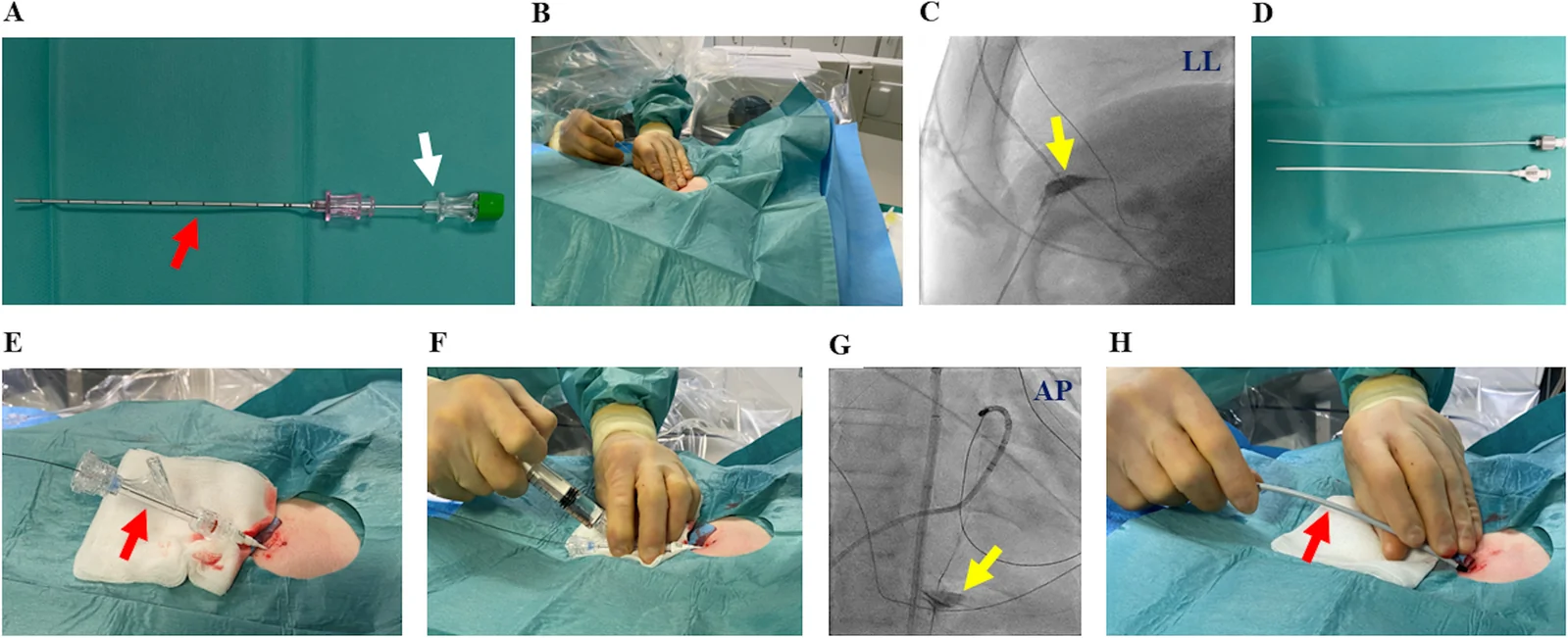
The standard workflow of NiN micropuncture technique for epicardial access. **A** The NiN micropuncture technique was performed using a coaxial system consisting of an 18 G outer standard pericardial access needle (red arrow) and a 21 G inner micropuncture needle (white arrow), **B** The outer needle was advanced incrementally through the subcutaneous tissue and diaphragm toward the pericardium under fluoroscopic monitoring. The C-arm was maintained in the 90° left lateral position, **C** A fluoroscopic view showing that the contrast was injected through a syringe to confirm entry into the pericardial space (yellow arrow) (90° left lateral view), **D** The dilation was performed using a 5 Fr dilator, **E** The Y connector was used (red arrow), **F** Contrast was injected to confirm that the sheath was in epicardial space, **G** A fluoroscopic view showing that the sheath was in the epicardial space (yellow arrow) (anteroposterior view), **H** An 8 Fr epicardial introducer sheath was inserted over the guidewire (red arrow). AP = anteroposterior; LL = left lateral

Epicardial access was performed in all cases using a NiN micropuncture technique via a subxiphoid approach. The skin puncture site was located approximately 1–2 cm inferior to the xiphoid process and about 1 cm left of the midline. The NiN micropuncture technique was performed using a coaxial system consisting of an 18 G outer standard pericardial access needle (Angiokard Medizintechnik) and a 21 G inner micropuncture needle (MAK-NV™, Merit Medical) (Fig. 1A). After infiltration of local anesthetic, the outer needle was advanced incrementally through the subcutaneous tissue and diaphragm toward the pericardium under fluoroscopic monitoring (Fig. 1B). A true 90° left lateral (LL) fluoroscopic projection was used from the beginning of needle advancement to aid visualization of the needle trajectory in relation to the cardiac silhouette. The outer needle was positioned immediately adjacent to the cardiac silhouette without intentional penetration of the pericardial sac. The inner micropuncture needle was then advanced gradually through the outer needle beyond the tip of the outer needle while continuous gentle aspiration was applied. Advancement was stopped immediately after suspected pericardial entry to avoid myocardial penetration. Entry into the pericardial space was suggested by loss of resistance and/or aspiration of a small amount of clear pericardial fluid. Correct positioning was confirmed by injection of a small volume of contrast medium (approximately 1–2mL) through the inner needle, demonstrating free dispersion within the pericardial space (Fig. 1C). Once pericardial access was obtained, a soft, atraumatic 0.018-inch guidewire (Nitrex™, Medtronic) was threaded through the microneedle into the pericardial space. Over this guidewire, dilation was performed using a 5 Fr dilator within the micropuncture kit (Merit MAK™, Merit Medical; Fig. 1D). Subsequently, a Y connector was used (Fig. 1E) and contrast was injected to confirm that the sheath was in the epicardial space under the anteroposterior fluoroscopic view (Fig. 1F and G). Thereafter, the thin wire was removed and replaced by a standard 0.032-inch guidewire, over which the 5 Fr dilator was exchanged for a standard 8 Fr introducer sheath (Fig. 1H).

After epicardial access was obtained, epicardial three-dimensional mapping was performed. When regions of interest were identified on the epicardial surface, coronary angiography was performed to ensure a safe distance from coronary arteries before ablation. Radiofrequency (RF) energy was delivered epicardially to target scar-related reentrant circuits or VT exit sites, as guided by substrate mapping and inducible VT morphology. Epicardial RF ablation was performed using an irrigated RF catheter. Power settings ranged generally from 30 to 40 W, with lesion duration of approximately 20–40 s according to substrate characteristics and anatomical considerations. Standard saline irrigation was used. When the epicardial target was located within 5 mm of the coronary artery, epicardial ablation was abandoned, and ablation was performed only from the endocardial side. In addition, phrenic nerve location was identified via high-output pacing. The phrenic nerve was monitored during epicardial ablation, especially when lesions were delivered near the course of the right phrenic nerve (along the right pericardial border) or left phrenic nerve (lateral to the left atrium). In case of close proximity of the ablation site to the phrenic nerve, fluid was injected into the pericardium in order to avoid phrenic damage. At the end of the procedure, lidocaine (50–100 mg) and steroids (equivalent to 150–300 mg of prednisolone) were administered into the pericardial space. Initially, a 6 F pigtail catheter was left in the pericardial space overnight for monitoring and removed next morning. In the later period, the pigtail catheter was removed immediately at the end of the procedure in order to improve patient comfort and potentially reduce post-procedural inflammatory symptoms.

### Antithrombotic management

Periprocedural antithrombotic management was performed as follows.

Vitamin K antagonists were continued within the therapeutic INR range (2.0–3.0), while direct oral anticoagulants were withheld on the morning of the procedure. Antiplatelet therapy was continued without interruption.

During the ablation procedure, unfractionated heparin was administered to maintain an activated clotting time (ACT) > 300 s. In patients who underwent epicardial procedures only without an endocardial approach, epicardial access was obtained before the initiation of systemic anticoagulation. In patients undergoing both endocardial and epicardial procedures, epicardial access was obtained after endocardial access without reversal of anticoagulation with protamine.

Oral anticoagulation was resumed the following morning after the procedure.

### Statistical analysis

All statistical analyses were performed using JMP 19 software (SAS Institute). Continuous variables were presented as medians with interquartile ranges, while categorical variables were expressed as counts and percentages. Exact binomial confidence intervals (Clopper–Pearson method) were calculated for proportions with zero or complete events.

## Results

### Patient characteristics

A total of 40 patients were included. Baseline characteristics are summarized in Table 1. The median age was 64 (52, 70) years and 23% of patients were female. Ischemic cardiomyopathy was present in 20% of patients. The median left ventricular ejection fraction was 42 (32, 48) %. No patient had a history of prior cardiac surgery, previous pericarditis, or previous epicardial ablation. Patients with expected severe pericardial adhesions were generally not considered candidates for percutaneous epicardial access at our institution.

**Table 1:** Baseline characteristics of the patients

Variables	All patients (*n* = 40)
Age (years)	64 (52, 70)
Female, n (%)	9 (23)
Body mass index (kg/m^2^)	26.9 (25.1, 29.8)
Underlying cardiomyopathy	
Ischemic cardiomyopathy, n (%)	8 (20)
Nonischemic cardiomyopathy, n (%)	32 (80)
Dilated cardiomyopathy, n	17
Myocarditis, n	5
Arrhythmogenic right ventricular cardiomyopathy, n	2
Cardiac sarcoidosis, n	1
Others, n	7
ICD or CRT-D, n (%)	34 (85)
Prior procedures	
Previous VT ablation, n (%)	26 (65)
Previous endocardial-only VT ablation, n (%)	26 (65)
Previous epicardial ablation, n (%)	0 (0)
Previous cardiac surgery, n (%)	0 (0)
Comorbidities	
Arterial hypertension, n (%)	18 (45)
Dyslipidemia, n (%)	20 (50)
Diabetes mellitus, n (%)	6 (15)
Atrial fibrillation, n (%)	12 (30)
Prescribed medications	
AAD, n (%)	28 (70)
Beta-blocker, n (%)	34 (85)
ACEI, ARB, or ARNI, n (%)	30 (75)
Antithrombotic drugs, n (%)	28 (70)
Laboratory data	
NT-proBNP (ng/L)	519 (269, 1446)
CRP (mg/L)	1.5 (0.8, 13.2)
Hb (g/dL)	13.7 (12.8, 14.9)
Echocardiographic parameters	
LVEDD (mm)	59 (53, 64)
LVEF (%)	42 (32, 48)
LAVI (mL/m^2^)	38 (31, 43)
Moderate or severe MR, n (%)	6 (15)

*AAD*, antiarrhythmic drug; *ACEI,* angiotensin converting enzyme inhibitor; *ARB,* angiotensin II receptor blocker; *ARNI,* angiotensin receptor-neprilysin inhibitor; *CRP,* C-reactive protein; *CRT-D,* cardiac resynchronization therapy-defibrillator; *Hb,* hemoglobin; *ICD,* implantable cardioverter defibrillator; *LAVI,* left atrial volume index; *LVEDD,* left ventricular end-diastolic diameter; *LVEF,* left ventricular ejection fraction; *MR,* mitral regurgitation; *NT-proBNP,* N-terminal pro-brain natriuretic peptide; *VT,* ventricular tachycardia

### Procedural details

Procedural details are shown in Table 2. Nurse-administered propofol sedation was used in 37 patients (93%). No patient required conversion from sedation to general anesthesia. No clinically relevant airway events or movement-related problems affecting epicardial access occurred. Epicardial access using the NiN micropuncture technique was successful in all 40 patients (100% success rate of pericardial entry and sheath placement). In 37 patients (93%), a single needle puncture attempt was sufficient to access the pericardial space on the first pass. The remaining 3 patients required a second attempt due to initial misalignment or minor adjustments. None of the cases required conversion to the conventional large-bore needle technique or surgical cutdown; the micropuncture method alone was adequate for all patients. The time from skin puncture to successful sheath insertion was short (generally less than 5 min for the access step in most cases), though total procedure durations varied depending on mapping complexity. Median total procedure time was 141 (120, 168) minutes. Median fluoroscopy time was 10 min; however, the actual fluoroscopy time required for epicardial access was substantially shorter and was estimated to be less than 2 min in most cases.

**Table 2:** Procedural characteristics

Variables	All patients (*n* = 40)
Deep sedation used, n (%)	37 (93)
Total procedure time, minutes	141 (120, 168)
Fluoroscopy time, minutes	10 (8, 14)
Dose [cGy·cm^2^]	596 (373, 885)
Epicardial access success, n (%)	40 (100)
First-pass needle success, n (%)	37 (93)
Needle puncture attempts	1 (1, 2)
90° left lateral view used, n (%)	40 (100)
Epicardial mapping performed, n (%)	40 (100)
Epicardial RF ablation delivered, n (%)	38 (95)
Combined endocardial plus epicardial ablation, n (%)	14 (35)
Acute VT non-inducibility achieved, n (%)	34 (85)

*RF,* radiofrequency; *VT,* ventricular tachycardia

Epicardial access was successfully achieved in all patients (100%; 95% confidence interval [CI], 91.2–100%). In 38 of 40 patients (95%), significant VT substrate or inducible VT circuits were identified on the epicardial surface and were targeted with ablation. In the other 2 patients (5%), epicardial mapping was performed but did not reveal an obvious arrhythmogenic substrate (in one case, the VT origin was revised to be intramural septal; in another, the arrhythmia was non-inducible on the day of the procedure). Nonetheless, even in those cases, epicardial access was still useful for pacing and verification of the absence of epicardial late potentials. Fourteen patients (35%) underwent both epicardial and endocardial ablation in the same session. Among these patients, seven (50%) had undergone previous endocardial VT ablation. The acute procedural success in terms of arrhythmia treatment was high: at the end of the procedure, 34 patients (85%) had no inducible VT with programmed stimulation, while 6 patients had non-clinical or slower VTs still inducible but were left due to either non-sustained nature or proximity to critical structures. Overnight drainage was performed in the earlier period (24 patients, 60%). In those patients, drain output was minimal (often < 50 mL of serous fluid) and the drain was removed without issues until the day after the procedure. Immediate drain removal was adopted in the later period.

### Acute safety outcomes

Table 3 summarizes the acute safety outcomes. No major access-related complications were observed (0%; 95% CI, 0–8.8%). In particular, no patients developed a large pericardial effusion during or after the procedure that required emergency pericardiocentesis or surgical intervention. Importantly, no patients required urgent cardiac surgery or blood transfusion due to the epicardial access. In addition, there were no erroneous punctures of abdominal viscera or the internal mammary vessels likely owing to the anterior trajectory, mindful needle advancement, and possibly the small needle caliber.

**Table 3:** Procedure-related complications

Variables	All patients (*n* = 40)
Cardiac tamponade requiring intervention, n (%)	0 (0)
Coronary artery injury, n (%)	0 (0)
Emergency cardiac surgery, n (%)	0 (0)
Abdominal organ injury, n (%)	0 (0)
Phrenic nerve injury, n (%)	0 (0)
Pneumothorax, n (%)	0 (0)
Complete atrioventricular block, n (%)	1 (2.5)
Procedure-related death, n (%)	0 (0)

The only significant adverse event in our cohort was a complete atrioventricular block, which occurred in a case with an inferoseptal scar-related VT. During epicardial ablation near the basal inferoseptal region (close to the atrioventricular groove), the patient developed sudden complete atrioventricular block, which persisted despite prompt cessation of energy delivery. The patient was hemodynamically stable with backup pacing from the implantable cardioverter defibrillator and no further invasive procedures were required. This complication was related not to epicardial access technique but to radiofrequency application. Another patient experienced a brief episode of non-sustained VT during needle advancement, likely due to mechanical irritation of the epicardium, which terminated spontaneously.

All 40 patients were discharged from the hospital without any procedure-related disability. There were no procedure-related deaths.

## Discussion

### Main findings

In this single-center study of 40 patients, we found that using a NiN micropuncture technique for epicardial access in VT ablation is highly feasible and appears to be acutely safe. All patients (100%) in our series were successfully accessed epicardially with NiN micropuncture technique, and no access-related major complications were observed.

### Feasibility and safety considerations

Our complication rates compare very favorably with previously reported complication rates on epicardial access using conventional large-bore needles. Previous studies using the standard 17–18 G Tuohy needle have reported that major complications occurred in roughly 3–10% of all cases with epicardial VT ablation [7–9]. Our experience aligns with emerging evidence that NiN micropuncture technique can reduce the risk of serious complications [5, 6]. One of the novel aspects of our approach was the use of the LL fluoroscopic view. Traditional epicardial access was often performed with the needle directed toward the inferior epicardial space under anteroposterior fluoroscopic view, which tended to access the epicardial space easily but had the risk of injury to abdominal organs. In our practice, the LL fluoroscopic view helps ensure the needle remains in the anterior plane and provides immediate feedback on depth. In other words, the anterior subxiphoid trajectory used in combination with LL fluoroscopy allows improved depth control and may reduce the risk of injury to abdominal organs compared with more inferior or posterior approaches. Another important aspect of our workflow was that the majority of procedures were performed under deep sedation. This is noteworthy because epicardial access is often perceived as requiring general anesthesia due to concerns about patient discomfort or sudden movement during subxiphoid puncture [10]. The controlled, stepwise nature of the NiN micropuncture technique, together with careful local anesthesia and fluoroscopic guidance, allowed stable and predictable access even under deep sedation. Avoiding general anesthesia may shorten recovery time, reduce anesthesia-related risks, and improve overall procedural efficiency, particularly in high-volume VT ablation centers.

The occurrence of complete atrioventricular block in one patient deserves some discussion, as it illustrates a risk of epicardial ablation that is not eliminated by the choice of needle. Complete heart block during VT ablation is relatively rare, especially from the epicardial side, but it can occur if ablation lesions affect the atrioventricular node or His bundle (for example, via collateral circulation or proximity to the penetrating bundle). In our case, the patient’s critical VT substrate lay near the inferoseptal region, and despite careful precautions, an epicardial RF application in that area led to AV block. This complication highlights that even with a perfectly executed access and no mechanical complications, the ablation itself must be approached cautiously when near sensitive structures.

### Endocardial and epicardial ablation strategy

In our cohort, combined endocardial and epicardial ablation was performed in 14 patients (35%). This reflects our individualized procedural strategy rather than a limitation of the access technique. Among patients undergoing combined procedures, 50% had undergone previous endocardial VT ablation.

At our institution, the decision to perform a combined endocardial–epicardial procedure or a staged approach is based on several factors, including the suspected substrate location, previous ablation history, procedural complexity, and the anticipated need for prolonged systemic anticoagulation during left ventricular mapping. In selected patients, epicardial access is performed after previous endocardial ablation failure or VT recurrence despite an endocardial approach.

A staged strategy may also be considered to minimize procedural complexity and bleeding risk associated with combined procedures requiring systemic anticoagulation. Conversely, when pre-procedural imaging, electroanatomical mapping, or clinical characteristics strongly suggest an epicardial substrate, combined endocardial and epicardial ablation may be performed during the same session.

Therefore, the proportion of combined procedures in this study reflects our institutional workflow and patient selection rather than a limitation of the NiN epicardial access technique.

### Comparison with other techniques or devices

Recent studies have demonstrated the safety and efficacy of CO_2_-assisted epicardial access techniques [11–13]. These approaches may improve safety by creating a pericardial space prior to needle advancement. However, they require additional equipment and procedural steps. In contrast, the NiN technique is simple, widely applicable, and does not require adjunctive tools. In addition, Novel devices such as curled-tip guidewire systems have also been introduced [14]. While promising, these technologies are not yet widely available. The NiN approach, by contrast, utilizes standard equipment and may therefore be more readily adopted.

### Strengths and limitations

This study has several important strengths. This is one of the largest clinical series evaluating a NiN micropuncture technique for epicardial access specifically in patients undergoing VT ablation. Our study provides a systematic assessment of procedural feasibility and acute safety in a consecutive cohort of 40 patients. Second, all epicardial access procedures were performed using a consistent workflow, including a standardized fluoroscopic strategy with early left lateral projection, which reduces procedural variability and allows detailed evaluation of the technique. Third, the NiN approach was successfully performed under nurse-administered propofol sedation in the majority of patients, suggesting that this technique may facilitate epicardial access without routine dependence on general anesthesia. This may be particularly relevant in contemporary VT ablation practice, where minimizing procedural complexity and improving patient recovery are increasingly important.

Several limitations should be acknowledged. First, this was a small, retrospective, single-center observational study. Second, all procedures were performed by a single highly experienced operator, which limits generalizability and may introduce operator-related bias. Third, no contemporaneous conventional needle control group was available because all epicardial access procedures during the study period were performed using the NiN technique. Therefore, the present findings should be interpreted as supportive of feasibility rather than evidence of superiority. Fourth, the study population did not include patients with prior cardiac surgery, pericarditis, or known significant pericardial adhesions; therefore, applicability in these challenging populations remains uncertain. Fifth, the analysis focused on acute procedural outcomes. Long-term clinical outcomes, including VT recurrence, post-procedural pain, and recovery time, were not systematically evaluated. Sixth, although no major complications occurred, the confidence intervals around zero-event outcomes remain wide because of the limited sample size. Seventh, detailed procedural metrics, including access duration, number of contrast injections, and needle redirection events, were not prospectively collected and could not be reliably analyzed retrospectively. Finally, prospective multicenter studies comparing NiN with conventional epicardial access techniques are required.

## Conclusions

In this retrospective single-center study, epicardial access for VT ablation using a NiN micropuncture technique was feasible and associated with no observed major access-related complications in a selected cohort. The technique provides a standardized approach for epicardial access; however, comparative studies are required to determine whether it offers advantages over conventional epicardial access methods.

## Data Availability

The data that support the findings of this study are available from the corresponding author upon reasonable request.

## Abbreviations and acronyms

LL: left lateral
NiN: needle-in-needle
VT: ventricular tachycardia
